# Effects of select copper sources at minimum supplementation levels on nutrient content, off-colors, and blemishes in canned pet food

**DOI:** 10.1093/tas/txac036

**Published:** 2022-03-30

**Authors:** Amanda N Dainton, Dana J Tomlinson, Charles Gregory Aldrich

**Affiliations:** 1 Department of Grain Science & Industry, Kansas State University, Manhattan, KS 66506, USA; 2 Zinpro Corporation, Eden Prairie, MN 55344, USA

**Keywords:** blemish, canned pet food, color, copper, vitamin E, wet pet food

## Abstract

In the previous research, super-fortification with copper decreased vitamin E content and darkened canned pet food, which prevented the analysis of black blemishes reported in commercial products. The pet food industry has linked these blemishes, which may be concerning to pet owners, to copper supplementation. The objective of this study was to determine the effect of different copper sources included at minimum recommended levels on nutrient content, color, and blemishes in canned pet food. Treatments were arranged in a 2 × 3 + 1 factorial, with 2 levels of copper supplementation [6 and 12 mg/kg dry matter (DM)], 3 copper sources (CG = copper glutamate, CA = copper amino acid complex, and CS = copper sulfate), and a control with no added copper (NC). Diets were analyzed for macronutrients (moisture, crude protein, crude fat, crude fiber, and ash) and micronutrients (calcium, phosphorus, potassium, sodium, magnesium, sulfur, iron, copper, manganese, zinc, and vitamin E). Color was quantified with a CIELAB color space colorimeter wherein L* values closer to 100 represented lighter products and more positive a* and b* values indicated redder and yellower products, respectively. Blemishes were enumerated and their surface area quantified with ImageJ software. Data were analyzed as a general linear mixed model with the fixed effect of treatment and the random effect of production day. *P*-values less than 0.05 were considered significant. The 12 mg/kg DM treatments (average 14.19 mg/kg DM) contained the highest (*P *< 0.05) level of copper, followed by 6 mg/kg DM treatments (average 7.59 mg/kg DM) and then NC (0.00 mg/kg DM). Addition of copper decreased (*P* < 0.05) vitamin E content, except for NC and CS12 which were similar (*P* > 0.05; average 111.89 mg/kg DM). Lightness (average L* 63.66) was not affected (*P* > 0.05) by the treatments. Adding copper decreased (*P* < 0.05) redness, with higher (*P* < 0.05) a* values for CG6 (9.55) vs. CA6 and CS6 (average 8.50). Yellowness also decreased with the addition of copper, except for CG6 which was similar (*P* > 0.05) to NC (average 18.70). However, CG6 and CG12 (average 4.05 blemishes/slice of food) contained more (*P* < 0.05) blemishes than CA6, CS6, and CS12 (average 0.97 blemishes/slice of food). Minimal levels of supplemental copper from CG may enhance overall color preservation but could increase blemish occurrence. No disadvantage was observed for CA vs. CS, indicating that CA could be exchanged for CS in formulations.

## INTRODUCTION

Copper must be supplemented in commercial diets for pets as it is an essential nutrient for dogs and cats and other ingredients in the formulation may not provide enough to meet an animal’s requirements (Ferreira et al., 2005). The Association of American Feed Control Officials (AAFCO) puts forth recommendations for the level of nutrients in foods for dogs and cats sold in the United States. Their recommendations for copper are 7.3 mg/kg dry matter (DM) for adult dogs and 5.0 mg/kg DM for adult cats (AAFCO, 2018). Traditionally, copper oxide was used as the copper source in pet foods, but it was found to be biologically unavailable in chickens (Baker et al., 1991) and growing cattle (Kegley and Spears, 1994). After this discovery, copper sulfate became the most used form of supplemental copper in companion animal diets. Shortly after this change, pet food companies noticed discoloration in thermally processed low-acid pet food, commonly referred to as canned pet food. This discoloration was characterized as an overall darkening and appearance of small black spots particularly in light-colored meat-based foods (chicken, turkey, etc.; Shields, 1998). These differences in color could be perceived as adulterated or spoiled product by the pet owner, leading to eroded consumer confidence.

Copper is known to function as an oxidizing agent in food reactions and is reduced from Cu^2+^ to Cu^+^. The electron donor for this reaction can come from many different ingredients, such as lipids or proteins. Copper-catalyzed lipid oxidation has been observed in simplistic models, such as emulsions containing canola oil (Osborn-Barnes and Akoh, 2003) or soy lecithin (Wang and Wang, 2008) and during storage of rapeseed oil (Andersson and Lingnert, 1998), but results are not conclusive in meat and animal-based products. Higher levels of copper were correlated to lipid oxidation and yellow-brown discoloration in salted cod (Lauritzsen et al., 1999) but did not result in development of rancid odors in Atlantic mackerel (Maestre et al., 2011) or increased oxidation levels in turkey breast (Salih et al., 1989). Limited research regarding the effects of copper on protein oxidation has demonstrated decreased protein functionality and increased red hues in minced cuttlefish (Thanonkaew et al., 2006). However, none of these mechanisms have been determined to cause the discoloration observed in canned pet foods.

One previous study addressed the inclusion of different copper sources in canned pet foods and found that treatments supplemented with copper were darker, less red and yellow, and exhibited decreased vitamin E levels (Dainton et al., 2021b). Organic/chelated copper sources were included in the study to determine if complexed copper protected the mineral from reacting with other ingredients. In that work copper was supplemented at high levels (60 and 300 mg/kg DM) and the foods were too dark for detection of black blemishes. Other differences, specifically lightness (L*), redness (a*), and vitamin E content were not distinguishable between the three copper sources tested. Therefore, the objective of this experiment was to determine the effects of organic and inorganic copper sources at minimally supplemented levels (AAFCO, 2018) on nutrient content, CIELAB color space values, and blemishes in a thermally processed canned pet food. The hypothesis was that an organic copper source would influence vitamin E content, color, and blemish count less than an inorganic copper source.

## MATERIALS AND METHODS

Treatments were arranged in a 2 × 3 + 1 augmented factorial, with two copper premix levels (6 mg/kg DM or 12 mg/kg DM), three sources of copper [copper glutamate (CG), copper amino acid complex (CA), or copper sulfate (CS)], and a negative control with no copper premix. The copper glutamate consisted of single copper ions bound to single glutamate amino acids (Zinpro Corporation; Eden Prairie, MN). Copper amino acid complex was a mixture of single copper ions bound to one of 17 possible single amino acids (Availa-Cu; Zinpro Corporation; Eden Prairie, MN). The final copper source evaluated, copper sulfate, comprised of single copper ions bound to single sulfate groups. This yielded seven treatments: no copper supplementation (NC), copper glutamate supplementation at 6 mg/kg DM (CG6), copper glutamate supplementation at 12 mg/kg DM (CG12), copper amino acid complex supplementation at 6 mg/kg DM (CA6), copper amino acid complex supplementation at 12 mg/kg DM (CA12), copper sulfate supplementation at 6 mg/kg DM (CS6), and copper sulfate supplementation at 12 mg/kg DM (CS12). These levels were chosen to meet the minimum recommended copper allowances for adult dogs (7.3 mg/kg DM) and cats (5 mg/kg DM; AAFCO, 2018).

Copper premixes were made in advance of diet production and were formulated to provide 6 mg/kg DM at a 0.25% inclusion and 12 mg/kg DM at a 0.50% inclusion (Table 1). Sources of copper (Zinpro Corporation, Eden Prairie, MN) were ground using a mortar and pestle before mixing with corn starch (Fairview Mills, Seneca, KS) in a table-top planetary mixer (KitchenAid Artisan, St. Joseph, MI) for 5 min. No other minerals were supplemented for a nutritional outcome in an effort to isolate the effect of copper supplementation and minimize confounding variables. The copper premixes were exchanged for whole white rice. Mechanically deboned chicken meat (CJ Foods, Inc., Bern, KS) was also ground through a 7 mm circular die plate (Weston Pro Series #32, Southern Pine, NC) before diet production.

**Table 1.** Ingredient composition of thermally processed canned pet food^1^ containing different copper sources at minimum levels to meet AAFCO^2^ recommendations

Ingredient, % as-is	NC	CG6	CG12	CA6	CA12	CS6	CS12
Mechanically separated chicken	55.10	55.10	55.10	55.10	55.10	55.10	55.10
Water	38.35	38.35	38.35	38.35	38.35	38.35	38.35
White rice	3.00	2.75	2.50	2.75	2.50	2.75	2.50
Carrageenan	1.00	1.00	1.00	1.00	1.00	1.00	1.00
Guar gum	1.00	1.00	1.00	1.00	1.00	1.00	1.00
Potassium chloride	0.50	0.50	0.50	0.50	0.50	0.50	0.50
Spray-dried egg white	0.50	0.50	0.50	0.50	0.50	0.50	0.50
Soybean oil	0.50	0.50	0.50	0.50	0.50	0.50	0.50
Vitamin premix^3^	0.05	0.05	0.05	0.05	0.05	0.05	0.05
CG copper premix^4^	–	0.25	0.50	–	–	–	–
CA copper premix^5^	–	–	–	0.25	0.50	–	–
CS copper premix^6^	–	–	–	–	–	0.25	0.50

NC = no added copper source; CG6 = 6 mg/kg DM of added copper from copper glutamate; CG12 = 12 mg/kg DM of added copper from copper glutamate; CA6 = 6 mg/kg DM of added copper from copper amino acid complex; CA12 = 12 mg/kg DM of added copper from copper amino acid complex; CS6 = 6 mg/kg DM of added copper from copper sulfate; CS12 = 12 mg/kg DM of added copper from copper sulfate.

AAFCO = Association of American Feed Control Officials.

One kg of vitamin premix supplies 17,163,000 IU vitamin A, 920,000 IU vitamin D_3_, 79,887 IU vitamin E, 22.0 mg vitamin B_12_ (cobalamin), 4,719 mg vitamin B_2_ (riboflavin), 12,186 mg vitamin B_5_ (d-pantothenic acid), 14,252 mg vitamin B_1_ (thiamin), 64,730 mg vitamin B_3_ (niacin), 5,537 mg vitamin B_6_ (pyridoxine), 720 mg vitamin B_9_ (folic acid), and 70.0 mg vitamin B_7_ (biotin).

500 g of CG copper premix contains 1.0292 g of copper glutamate and 498.9708 g of cornstarch.

500 g of CA copper premix contains 2.5631 g of copper amino acid complex and 497.4369 g of cornstarch.

500 g of CS copper premix contains 1.0373 g of copper sulfate and 498.9627 g of cornstarch.

Experimental batches (*n* = 3) were produced over 3 days with each treatment made once daily. Water was heated in a pot to 40 °C, at which point chicken, white rice (Kroger, Manhattan, KS), spray-dried egg white (Rembrandt Foods, Okoboji, IA), soybean oil (Kroger, Manhattan, KS), potassium chloride (Bill Bar and Company, Overland Park, KS), vitamin premix (DSM, Heerlen, NL), and a copper premix (per protocol) were added. Guar gum (Danisco, Copenhagen, DE) and carrageenan (Danisco, Copenhagen, DE) were added once the mixture reached 60 °C. Cans (300 × 407; Freund Container & Supply, Lisle, IL) were filled with 400 g of each diet and lids (300; Freund Container & Supply, Lisle, IL) were seamed (Dixie Seamer, 91118; Athens, GA). Cans from all treatments prepared on the same day were placed into a retort (Dixie, 00-43; Athens, GA) and processed together at 121 °C and 144.79 kPa until a lethality of 8 min was reached. The first can of each treatment contained a thermocouple (Ecklund-Harrison Technologies, Fort Myers, FL) connected to a data logger (TechniCAL, LLC, Metairie, LA) to record internal product temperature. These collected data were recorded every 15 s (0.25 min) with software (CALSoft 5; TechniCAL LLC, Metairie, LA) for lethality calculations utilizing standard values for *Clostridium botulinum* (Eq. 1; Singh and Heldman, 2014). Briefly, the software recognized *T*_C_(*t*) as the internal can temperature at time *t* and Δ*t* as the length of time between temperature recordings. Processing was deemed “complete” when the last can containing a thermocouple reached a lethality value of 8 min. After processing was complete, the retort was drained and filled with municipal water for 20 min, twice, before cans were removed from the retort.


$$\begin{array}{l} Lethality=\int 10\frac{T_{C}\left(t\right)-121.11\;{}^{\circ }C}{10\;{}^{\circ }C}\Delta t, \end{array}$$ (1)

Three cans from each day and treatment were composited and subsampled for analysis of moisture (AOAC 930.15), crude protein (AOAC 990.03), crude fat (AOAC 954.02), crude fiber (AOCS Ba 6a-05), and ash (AOAC 942.05) at a commercial laboratory (Midwest Laboratories, Omaha, NE). The same samples were also analyzed for sulfur, phosphorus, potassium, magnesium, calcium, sodium, iron, manganese, copper, and zinc (AOAC 985.01; Midwest Laboratories, Omaha, NE). Vitamin E was determined as alpha tocopherol (NP Analytical Laboratories, St. Louis, MO). Samples that were below the detection minimum were treated as zero values. Three cans from each day and treatment were sliced into four sections and each slice surface was analyzed with a colorimeter (CR-410 Chroma Meter, Konica Minolta, Chiyoda, Tokyo, JP) for color components. Color components were brightness (L*) and measurement on the red/green (a*) and yellow/blue (b*) scales. Pictures were taken of slices with a manual digital camera (Nikon D3100; Melville, NY) for blemish enumeration and characterization (ImageJ, Rockville, MD). Blemishes were defined as small darkened areas visible to the human eye with those ≥ 1 mm^2^ in area being the most easily recognizable.

Results were analyzed as a one-way analysis of variance (ANOVA) with seven treatments (Marini, 2003). Data were analyzed as a generalized linear mixed model with diet as the fixed effect production day as the random effect (SAS 9.4; SAS Institute, Cary, NC). The experimental unit for statistical analysis was one complete and independent production of a dietary treatment for a total of three experimental units per dietary treatment. The rationale for the number of experimental units per dietary treatment was based on previous research on the nutritional and physical characteristics of canned pet food (Dainton et al., 2021a,  2021b) and extruded pet food (Donadelli et al., 2020, 2021; Alvarenga et al., 2021). Each response variable was tested for normality and homogeneity of variances. Data transformations were used as follows: copper, blemishes per slice, and blemishes ≤ 1 mm^2^ per slice were transformed with the square root function; crude fiber, ash, magnesium, sodium, and b* were transformed with the cube root function; and crude fat was transformed with the logarithm function. Fishers LSD tests were used to separate treatment means with differences at *P < *0.05. Data have been presented as least square means with a 95% confidence interval (in pre-transformation units where applicable).

## RESULTS

### Macronutrient and Micronutrient Content

Macronutrient content of the pet foods was not affected (*P* > 0.05; Table 2) by the treatments except for ash content (*P* = 0.0230). Ash content ranged from 5.20% DM (NC) to 6.34% DM (CA12). The treatments CA12, CS6, and CG12 (average 6.16% DM) contained 14.9% more (*P *< 0.05) ash than CG6 (5.36% DM) with CS12 and CA6 (average 5.88% DM) intermediate and not different (*P* > 0.05). Similarly, CS12 (5.94% DM) contained 14.2% more (*P* < 0.05) ash than NC (5.20% DM) with CA6 and CG6 (average 5.59% DM) intermediate and not different (*P* > 0.05). Otherwise, the average macronutrient contents were 75.5% moisture, 34.5% DM crude protein, 41.0% DM crude fat, and 0.59% DM crude fiber.

**Table 2.** Macronutrient content [least square mean (95% confidence interval)] of thermally processed canned pet food^1^ containing different copper sources at minimum levels to meet AAFCO^2^ recommendations.

Nutrient, %^3^	NC	CG6	CG12	CA6	CA12	CS6	CS12	*P*-value
Moisture	75.2 (74.1,76.3)	75.4 (74.3,76.5)	74.7 (73.6,75.8)	75.1 (74.0,76.2)	76.2 (75.1,77.3)	75.7 (74.6,76.8)	76.3 (75.2,77.4)	0.3441
	Dry matter basis							
Crude protein	34.0 (32.4,35.6)	33.2 (31.6,34.9)	34.2 (32.6,35.8)	35.1 (33.5,36.8)	35.9 (34.3,37.5)	34.7 (33.1,36.3)	34.5 (32.8,36.1)	0.3192
Crude fat	40.1 (38.1,42.0)	41.9 (39.9,44.0)	40.9 (38.9,42.9)	40.6 (38.6,42.6)	40.6 (38.6,42.6)	42.7 (40.7,44.9)	40.5 (38.5,42.5)	0.4523
Crude fiber	0.64 (0.43,0.93)	0.44 (0.27,0.66)	0.66 (0.43,0.94)	0.58 (0.37,0.84)	0.64 (0.41,0.91)	0.63 (0.41,0.91)	0.57 (0.37,0.84)	0.7671
Ash	5.20^c^ (4.78,5.64)	5.36^bc^ (4.94,5.81)	6.08^a^ (5.62,6.57)	5.81^abc^ (5.36,6.28)	6.34^a^ (5.86,6.84)	6.05^a^ (5.59,6.53)	5.94^ab^ (5.49,6.42)	0.0230

Least square means within the same row that do not share a common superscript are different (*P* < 0.05).

NC = no added copper source; CG6 = 6 mg/kg dry matter (DM) of added copper from copper glutamate; CG12 = 12 mg/kg DM of added copper from copper glutamate; CA6 = 6 mg/kg DM of added copper from copper amino acid complex; CA12 = 12 mg/kg DM of added copper from copper amino acid complex; CS6 = 6 mg/kg DM of added copper from copper sulfate; CS12 = 12 mg/kg DM of added copper from copper sulfate.

AAFCO = Association of American Feed Control Officials.

Three replicates per treatment.

All analyzed micronutrients were similar (*P* > 0.05) except for phosphorus (*P* = 0.0004; Table 3), calcium (*P* = 0.0004; Table 3), copper (*P* < 0.0001; Table 4), and vitamin E (*P* = 0.0104; Table 4). The average treatment contained 1.96 ± 0.103% DM potassium, 0.26 ± 0.028% DM sodium, 0.09 ± 0.015% DM magnesium, 0.63 ± 0.030% DM sulfur, 65.5 ± 4.10 mg/kg DM iron, and 34.3 ± 2.47 mg/kg DM zinc. Manganese was not supplemented and fell below the detection limit of 1 mg/kg as-is basis for all diets. Phosphorus and calcium were highest (*P* < 0.05) for CG12 (0.63% DM and 0.73% DM, respectively). The CA12 (0.57% DM) food contained more (*P* < 0.05) phosphorus than CG6 (0.48% DM), with CS12, NC, CS6, and CA6 all intermediate and not different (*P* > 0.05; average 0.51% DM) from either. The same treatment (CA12; 0.61% DM) also contained more (*P* < 0.05) calcium than CS12, CS6, and CA6, which were not different (*P* > 0.05; average 0.48% DM) from each other. The calcium level for NC was intermediate and not different (*P* > 0.05; 0.52% DM). However, it did contain more (*P* < 0.05) calcium than CG6 (0.42% DM), with CS12, CS6, and CA6 not different (*P* > 0.05; average 0.48% DM) from either.

**Table 3.** Dry matter basis micromineral content [least square mean (95% confidence interval)] of thermally processed canned pet food^1^ containing different copper sources at minimum levels to meet AAFCO^2^ recommendations.

Micromineral, %^3^	NC	CG6	CG12	CA6	CA12	CS6	CS12	*P*-value
Calcium	0.52^bc^ (0.44,0.61)	0.42^d^ (0.34,0.50)	0.73^a^ (0.64,0.81)	0.45^cd^ (0.40,0.54)	0.61^b^ (0.53,0.70)	0.47^cd^ (0.38,0.55)	0.51^cd^ (0.42,0.59)	0.0004
Phosphorus	0.52^bcd^ (0.48,0.57)	0.48^d^ (0.43,0.52)	0.63^a^ (0.58,0.68)	0.49^cd^ (0.45,0.54)	0.57^b^ (0.53,0.62)	0.51^cd^ (0.46,0.55)	0.53^bc^ (0.49,0.58)	0.0004
Potassium	1.88 (1.76,2.00)	1.91 (1.79,2.03)	1.91 (1.79,2.04)	1.97 (1.85,2.09)	2.03 (1.90,2.15)	1.93 (1.81,2.06)	2.05 (1.93,2.17)	0.3409
Sodium	0.26 (0.22,0.29)	0.29 (0.26,0.33)	0.27 (0.23,0.30)	0.25 (0.22,0.29)	0.27 (0.23,0.30)	0.25 (0.22,0.28)	0.27 (0.23,0.30)	0.4905
Magnesium	0.09 (0.07,0.12)	0.08 (0.06,0.10)	0.09 (0.07,0.12)	0.10 (0.07,0.12)	0.08 (0.06,0.11)	0.09 (0.07,0.12)	0.08 (0.06,0.11)	0.8189
Sulfur	0.62 (0.58,0.66)	0.61 (0.57,0.65)	0.63 (0.59,0.67)	0.63 (0.59,0.68)	0.63 (0.59,0.67)	0.63 (0.58,0.67)	0.65 (0.60,0.69)	0.9031

Least square means within the same row that do not share a common superscript are different (*P* < 0.05).

NC = no added copper source; CG6 = 6 mg/kg dry matter (DM) of added copper from copper glutamate; CG12 = 12 mg/kg DM of added copper from copper glutamate; CA6 = 6 mg/kg DM of added copper from copper amino acid complex; CA12 = 12 mg/kg DM of added copper from copper amino acid complex; CS6 = 6 mg/kg DM of added copper from copper sulfate; CS12 = 12 mg/kg DM of added copper from copper sulfate.

AAFCO = Association of American Feed Control Officials.

Three replicates per treatment.

**Table 4.** Dry matter basis trace mineral and vitamin E content [least square mean (95% confidence interval)] of thermally processed canned pet food^1^ containing different copper sources at minimum levels to meet AAFCO^2^ recommendations.

Nutrient, mg/kg^3^	NC	CG6	CG12	CA6	CA12	CS6	CS12	*P*-value
Iron	64.1 (58.6,69.7)	65.5 (60.0,71.1)	69.0 (63.5,74.6)	66.0 (60.5,71.6)	63.6 (58.0,69.2)	63.9 (58.3,69.4)	66.1 (60.6,71.7)	0.3401
Copper	0.00^c^ (−0.03,0.03)	7.58^b^ (6.73,8.49)	14.09^a^ (12.91,15.31)	7.64^b^ (6.78,8.55)	14.12^a^ (12.94,15.35)	7.51^b^ (6.66,8.41)	14.32^a^ (13.14,15.56)	<0.0001
Manganese	0.00	0.00	0.00	0.00	0.00	0.00	0.00	NA^4^
Zinc	34.9 (32.3,37.4)	32.9 (30.4,35.4)	38.3 (35.8,40.8)	34.3 (31.8,36.8)	33.2 (30.3,35.3)	33.8 (31.2,36.3)	32.8 (30.3,35.3)	0.0623
Vitamin E	112.72^a^ (101.07,124.37)	94.47^b^ (82.81,106.12)	92.67^b^ (81.02,104.32)	76.07^c^ (64.42,87.72)	91.84^bc^ (80.12,103.49)	92.95^b^ (81.30,104.60)	111.06^a^ (99.41,122.71)	0.0104

Least square means within the same row that do not share a common superscript are different (*P* < 0.05).

NC = no added copper source; CG6 = 6 mg/kg dry matter (DM) of added copper from copper glutamate; CG12 = 12 mg/kg DM of added copper from copper glutamate; CA6 = 6 mg/kg DM of added copper from copper amino acid complex; CA12 = 12 mg/kg DM of added copper from copper amino acid complex; CS6 = 6 mg/kg DM of added copper from copper sulfate; CS12 = 12 mg/kg DM of added copper from copper sulfate.

AAFCO = Association of American Feed Control Officials.

Three replicates per treatment.

NA = not applicable due to values below detection limits.

Copper targets were successfully met and were different (*P* < 0.0001; Table 4) among the treatments. Average copper content across copper sources for the 6 mg/kg DM added copper treatments was 7.59 ± 0.677 mg/kg DM and for the 12 mg/kg added copper treatments was 14.19 ± 0.877 mg/kg DM. The level of copper in NC was below the detection limit of 1 mg/kg as-is basis. Vitamin E content was altered (*P* = 0.0104) by the addition of copper. The NC treatment (112.72 mg/kg DM) contained more (*P* < 0.05) vitamin E than the copper-containing treatments except for CS12 (111.06 mg/kg DM), which was not different (*P* > 0.05). On an average, the CA6 treatment (76.07 mg/kg DM) contained 18.5% less (*P* < 0.05) vitamin E than CG6, CG12, and CS6 (average 93.36 mg/kg DM) with CA12 intermediate and not different (*P* > 0.05; 91.84 mg/kg DM) (Table 4).

### Overall Color and Blemishes

Differences across treatments were noted in a* (*P* < 0.0001) and b* (*P* < 0.0001) color values, but not L* (*P* = 0.1744; Table 5). Compared to NC, the copper-containing treatments were less (*P* < 0.05) red and yellow except for CG6, which was similar (*P* > 0.05) in yellow color. In general, treatments targeting 12 mg/kg of copper were less (*P* < 0.05) red and yellow, in addition to darker, than those supplemented with 6 mg/kg of copper. Only CA6 and CA12 were similar (*P* > 0.05) when supplementation levels from the same copper source were compared to each other. Within the 6 mg/kg DM supplementation level, CG6 was redder (*P* < 0.05) than CA6 and CS6, which were not different (*P *> 0.05). No differences (*P* > 0.05) were observed for yellow color. For the 12 mg/kg DM supplementation level, CG12 was redder (*P* < 0.05) than CA12 and CS12 was intermediate and not different (*P* > 0.05) from either. As with the 6 mg/kg DM supplementation treatments, the 12 mg/kg DM treatments were not different (*P* > 0.05) in terms of yellow color.

**Table 5.** Quantitative visual analysis of thermally processed canned pet food^1^ containing different copper sources at minimum levels to meet AAFCO^2^ recommendations.

Measurement^3^	NC	CG6	CG12	CA6	CA12	CS6	CS12	P-value
L*^4^	64.92 (62.90,66.93)	64.91 (62.89,66.93)	61.50 (59.49,63.52)	63.70 (61.69,65.72)	63.10 (61.08,65.12)	64.30 (62.29,66.32)	63.17 (61.15,65.19)	0.1744
a*^5^	10.58^a^ (9.89,11.27)	9.55^b^ (8.54,10.24)	8.12^cd^ (7.42,8.81)	8.32^c^ (7.63,9.02)	6.97^e^ (6.28,7.66)	8.67^c^ (7.98,9.37)	7.45^de^ (6.75,8.14)	<0.0001
b*^6^	19.22^a^ (18.38,20.08)	18.18^ab^ (17.37,19.00)	16.14^cde^ (15.39,16.89)	17.08^bcd^ (16.30,17.86)	16.04^de^ (15.30,16.80)	17.19^bc^ (16.41,17.99)	15.38^e^ (14.66,16.12)	<0.0001
Blemishes per slice	0.02^c^ (−0.12,0.39)	3.97^a^ (2.28,6.14)	3.57^a^ (1.97,5.63)	0.88^b^ (0.21,2.03)	1.87^ab^ (0.78,3.43)	0.70^b^ (0.12,1.74)	1.32^b^ (0.44,2.67)	0.0010
Blemishes ≥ 1 mm^2^ per slice	0.03^d^ (−0.03,0.21)	1.72^ab^ (0.98,2.62)	1.87^a^ (1.11,2.84)	0.39^c^ (0.10,0.89)	0.76^bc^ (0.31,1.41)	0.19^cd^ (0.02,0.57)	0.69^bc^ (0.26,1.31)	0.0006
Largest spot area, mm^2^	1.73	13.88	15.18	7.88	74.79	4.75	6.30	–
Average spot area, mm^2^	1.63	1.86	2.41	1.80	2.41	1.20	1.35	–

Least square means (95% confidence interval) within the same row that do not share a common superscript are different (*P* < 0.05).

NC = no added copper source; CG6 = 6 mg/kg dry matter (DM) of added copper from copper glutamate; CG12 = 12 mg/kg DM of added copper from copper glutamate; CA6 = 6 mg/kg DM of added copper from copper amino acid complex; CA12 = 12 mg/kg DM of added copper from copper amino acid complex; CS6 = 6 mg/kg DM of added copper from copper sulfate; CS12 = 12 mg/kg DM of added copper from copper sulfate.

AAFCO = Association of American Feed Control Officials.

Three replicates per treatment.

L* represents the lightness/darkness scale of color. L* values closer to 100 represent lighter colors, whereas L* values closer to 0 represent darker colors.

a* represents the red/green scale of color. More negative a* values represent greener color and more positive a* values represent redder color. a* values of 0 represent the absence of red or green color.

b* represents the yellow/blue scale of color. More negative b* values represent bluer color and more positive b* values indicate yellower color. b* values of 0 represent the absence of yellow or blue color.

**Figure 1.**
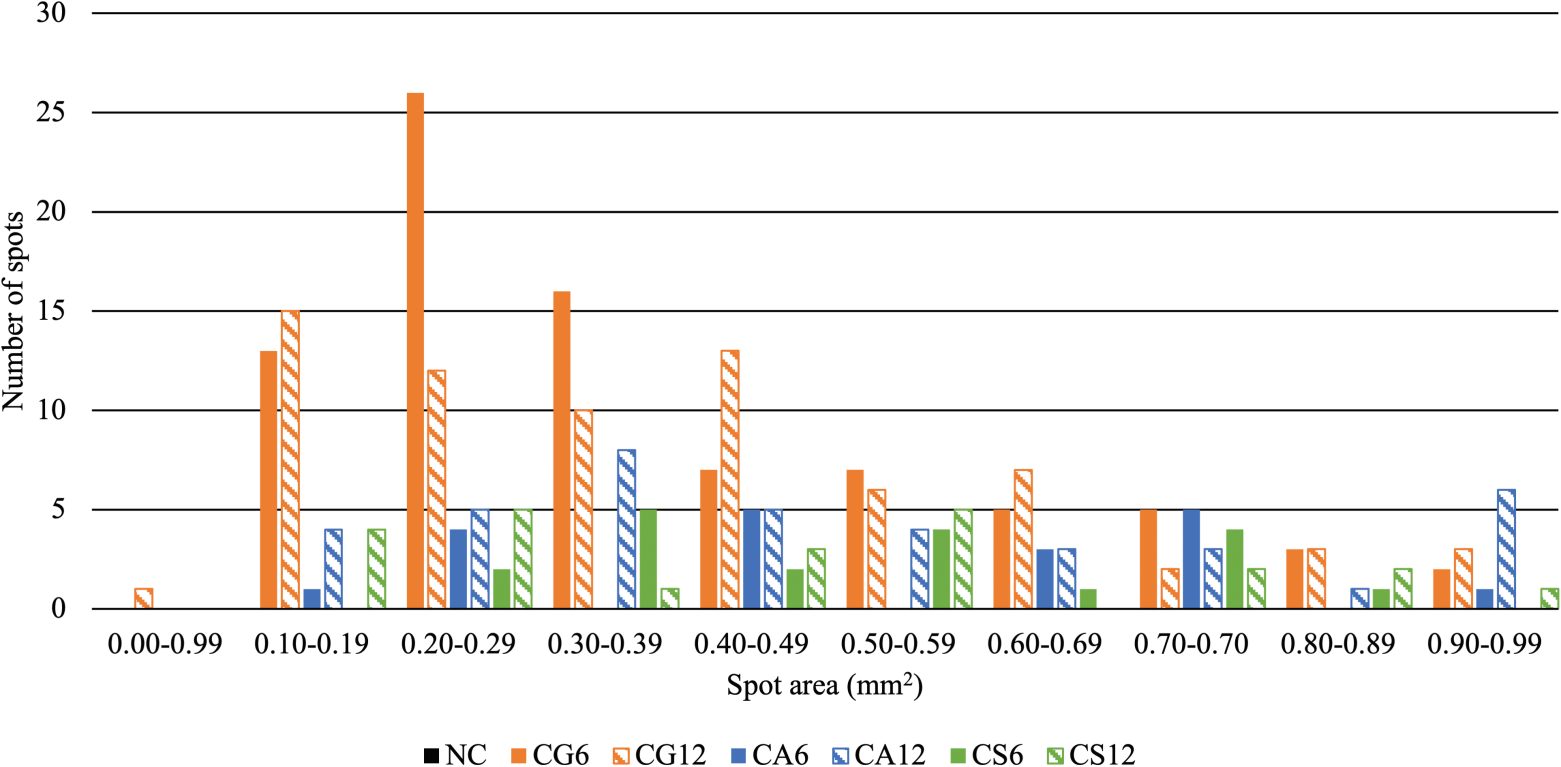
Distribution of spots < 1 mm^2^ in area found in thermally processed canned pet food^1^ containing different copper sources at minimum levels to meet AAFCO^2^ recommendations.

**Figure 2.**
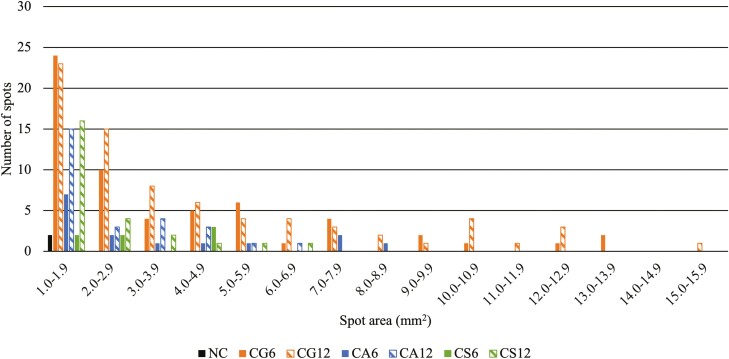
Distribution of spots  ≥ 1 mm^2^ and  < 16 mm^2^ in area found in thermally processed canned pet food^1^ containing different copper sources at minimum levels to meet AAFCO^2^ recommendations.

All copper-supplemented treatments contained more (*P* < 0.05) total blemishes per slice of product compared to NC (0.02). Within the copper-supplemented treatments, CG6 and CG12 (average 3.77) contained 289% more (*P* < 0.05) blemishes per slice than CA6, CS6, and CS12 (average 0.97) with CA12 (1.87) intermediate and not different (*P* > 0.05) When only blemishes > 1 mm^2^ in area were considered, CG12 (1.87) contained 156% more (*P* < 0.05) large blemishes per slice than CA12 and CS12 (average 0.73). Of the 6 mg/kg DM supplemented copper treatments, CG6 (1.72) contained 493% more (*P* < 0.05) large blemishes compared to CA6 and CS6 (average 0.29). The only treatment to contain similar (*P* > 0.05) levels of large blemishes compared to NC (0.03) was CS6 (0.19). The majority of spots for each treatment were smaller than 1 mm^2^ with most around 0.1–0.5 mm^2^ (Fig. 1). Most blemishes between 1 and 16 mm^2^ were between 1 and 2 mm^2^ and numbers decreased as blemish area increased (Fig. 2). Area of the largest blemishes observed in a treatment ranged from 1.73 mm^2^ (NC) to 74.79 mm^2^ (CA12). The average blemish size ranged from 1.20 mm^2^ (CS6) to 2.41 mm^2^ (CG12 and CA12; Table 5).

^1^NC = no added copper source; CG6 = 6 mg/kg DM of added copper from copper glutamate; CG12 = 12 mg/kg DM of added copper from copper glutamate; CA6 = 6 mg/kg DM of added copper from copper amino acid complex; CA12 = 12 mg/kg DM of added copper from copper amino acid complex; CS6 = 6 mg/kg DM of added copper from copper sulfate; CS12 = 12 mg/kg DM of added copper from copper sulfate.

^2^AAFCO = Association of American Feed Control Officials.

^1^NC = no added copper source; CG6 = 6 mg/kg DM of added copper from copper glutamate; CG12 = 12 mg/kg DM of added copper from copper glutamate; CA6 = 6 mg/kg DM of added copper from copper amino acid complex; CA12 = 12 mg/kg DM of added copper from copper amino acid complex; CS6 = 6 mg/kg DM of added copper from copper sulfate; CS12 = 12 mg/kg DM of added copper from copper sulfate.

^2^AAFCO = Association of American Feed Control Officials.

## DISCUSSION

In this experiment, three copper sources were included at two inclusion levels to determine if an organic copper source would induce fewer visual changes in a chicken-based formula compared to copper sulfate, the standard copper source used in canned pet food. The hypothesis was that copper chelation would make the copper less reactive, ultimately causing less change in product color and the development of black blemishes. Chelation has been suggested to decrease the reactivity of trace minerals and oxidation in comparison to other forms (Coelho, 2003). Copper glutamate was selected because copper-lysine-glutamate showed promise when supplemented at a 60 mg/kg level in a previous experiment (Dainton et al., 2021b). Copper amino acid complex was included to illustrate the differences when copper is chelated with specific amino acids vs. nonspecific amino acids. Both products are chelated at a one-to-one metal to amino acid ratio, but the specific vs. nonspecific amino acid complex may have affected processing differences.

### Macronutrient and Micronutrient Content

Ash content was the only macronutrient altered by the treatments. This was expected as moisture and crude fiber are not affected by oxidation and crude analysis of protein and fat may not be sensitive enough to detect differences. Ash analysis can account for calcium, phosphorus, potassium, sodium, magnesium, iron, copper, manganese, and zinc (National Research Council, 2006). Sulfur content may also contribute to ash analysis. As such, the differences in ash content do not directly mirror the differences in copper content and were very likely influenced by the measurable differences in calcium and phosphorus content. There are no recommended allowances for moisture, crude fiber, or ash for dog or cat diets. However, all seven diets in this experiment exceeded the minimum crude fat (5.5% DM and 9% DM) and crude protein (18% DM and 26% DM) recommendations for adult dogs (5.5% DM and 18% DM, respectively) and cats (9% DM and 26% DM, respectively; AAFCO, 2018).

Many of the experimental diets did not meet the mineral recommendations for adult dogs and cats (AAFCO, 2018). This was not unexpected as copper was the only mineral supplemented in the diets. This choice was made to minimize any confounding effects other minerals may have on the vitamin E and visual analyses. The only minerals that were different among the diets were calcium, phosphorus, and copper. The main source of calcium and phosphorus was likely the mechanically separated chicken. This ingredient is produced by removing any meat remaining on bones after cuts for human consumption were removed (Trindade et al., 2004). It is common that some bone is not fully separated from the meat and is incorporated into the processed ingredient. Reports of the calcium content in mechanically separated chicken are scarce but more common than reports of phosphorus content. Samples of Serbian mechanically separated chicken contained 0.068–0.090% calcium on an as-is basis (Tasić et al., 2017). Calcium content is affected by the chickens used to produce the ingredient. For example, Brazilian mechanically separated chicken from broilers contained 0.299% calcium as-is basis, whereas the same ingredient produced with spent hens contained 0.448% calcium as-is basis (Contreras-Castillo et al., 2008). Phosphorus is present in bone as hydroxyapatite, which also includes calcium (Case et al., 2011). This could explain why calcium levels and phosphorus levels followed similar relationships across the seven experimental diets. The mechanically separated chicken used to produce the experimental diets came 1–2 blocks in the same lot of meat. Therefore, it is unlikely that differences in ingredient production or source material caused the slight differences identified in the present experiment. Instead, the differences were likely caused by variability inherent to the chemical analyses. The calcium and phosphorus recommendations (AAFCO, 2018) for adult dogs (0.5% DM and 0.4% DM, respectively) and cats (0.6% DM and 0.5% DM, respectively) were not met for a few diets. This indicated that the observed differences may not have practical implications. Nevertheless, canned pet food formulators could increase the levels of calcium and phosphorus by replacing some of the mechanically separated chicken with a chicken ingredient that contains more bone, such as whole ground chicken.

Copper content was successfully altered by the addition of custom copper premixes. Levels used in this experiment were lower than previously examined in canned pet foods (Dainton et al., 2021b) and likely better represented the copper concentration found in commercial pet foods. Reports of copper content in commercial canned pet foods are limited. Copper content of canned food purchased in Turkey ranged from 4.91 mg/kg DM to 10.2 mg/kg DM for dogs and from 7.62 mg/kg DM to 16.6 mg/kg DM for cats (Duran et al., 2010). The canned cat food with a copper level of 7.62 mg/kg DM was manufactured in the United States. As such, the canned pet foods produced in the present experiment fall within these ranges and may be suitable models for commercial products. All seven experimental diets, except for NC, met the copper recommendation for adult dogs (7.3 mg/kg DM) and adult cats (5 mg/kg DM). This further supported the notion that supplemental copper should be included in a premix to create a complete and balanced diet.

Copper premixes were made in this experiment to uniformly disperse the copper supplementation throughout each batch. Supplemental vitamins and minerals are typically added to commercial pet foods as premixes to prevent the micronutrients from clumping, dispersing into the air, or adhering to the mixing equipment (Turlington, 2005). Consumption of inadequate or excess copper can be detrimental to pet health. Kittens do not grow as quickly (Doong et al., 1983) and queens take longer to become pregnant (Fascetti et al., 2000) if they do not consume enough copper. Some dog breeds, such as Labrador retrievers (Fieten et al., 2016) and Bedlington terriers (Forman et al., 2005; Haywood et al., 2016), are especially susceptible to overconsumption of copper and have issues with copper storage. However, cornstarch may not have been the ideal premix carrier. Examples of traditional grain-based carriers used by the pet food industry include rice hulls, wheat middlings, and various corn products (Turlington, 2005). Grain free carrier options may include soybean hulls and pea fiber (Pontious et al., 2018). The ideal premix carrier will flow easily and be similar in particle size to the microingredients contained within the premix (Turlington, 2005). The present experiment did not evaluate the particle size or flowability of the premixes created, though particle size of the copper sources was reduced with a mortar and pestle to mimic the cornstarch particle size. It is possible that using a premix carrier other than cornstarch would have yielded different results. Future experiments should use more common carriers, such as rice hulls or pea fiber, to better mimic a commercial canned pet food.

The vitamin E results were surprising. Previous research found that increased levels of trace minerals increased oxidation in pet food (Coelho, 2003). If that were the case, vitamin E could act as an antioxidant by reacting with copper and minimizing the oxidation of other nutrients. It was anticipated that the higher level of copper supplementation (12 mg/kg DM vs. 6 mg/kg DM) would result in a greater decrease in vitamin E content. Instead, CS12 contained more vitamin E than CS6 and vitamin E content was not different for CG6 vs. CG12 or CA6 vs. CA12. The lack of difference in vitamin E between NC and CS12 was also unexpected. This may have occurred due to the low level of copper supplementation; a previous study observed an average 15.36% reduction in vitamin E by adding a copper supplement targeting 60 or 300 mg/kg DM to a thermally processed canned pet food (Dainton et al., 2021b). Reduction of vitamin E due to copper supplementation in broiler chick feed was only observed after 10 days of storage (Luo et al., 2005; Lu et al., 2010). It is possible that copper’s effect on vitamin E is only evident at super-fortified levels or after prolonged storage. Furthermore, differences noted in the present experiment may simply be due to sampling or analytical variation. For example, vitamin E content was numerically 15.77 mg/kg DM higher with the higher inclusion of CA vs. the lower level. The standard deviation for vitamin E content of CA6 (±20.25 mg/kg DM) was greater than for CA12 (±6.49 mg/kg DM) and prevented differentiation between the treatments. Additionally, it would have aided in understanding the effects of copper source and supplementation level on vitamin E if the pre-retort processing vitamin E level was known.

### Overall Color and Blemishes

The present study showed that food darkening was not affected by copper supplementation, but increased copper concentration resulted in decreased redness and yellowness of product. This agrees with the reduction in a* values with higher levels of copper supplementation and supports that higher copper levels often result in darkened canned pet foods (Dainton et al., 2021b). In the previous study, differences in yellow color changes were not quantifiable at higher copper concentrations; the current study supports the notion that the previous over-fortification of copper masked differences in a* and b* values between supplementation levels and sources. These color shifts were small, especially the yellow-blue shift, but products could be differentiated by the human eye with proper training. Other researchers have linked copper to increased yellow hues and lipid oxidation (Lauritzsen et al., 1999) and to decreased red hues and protein oxidation (Thanonkaew et al., 2006). The decrease in red hues observed in the present experiment suggest a protein oxidation reaction with copper. Analyses to identify protein oxidation could include functionality tests, such as protein solubility, or quantification of carbonyls (Levine et al., 1990). In those instances, decreased protein solubility or greater concentration of carbonyls would indicate increased protein oxidation.

This is the first study to quantify and characterize blemishes in thermally processed canned pet foods. Theoretically, incidence of blemishes would indicate that the added copper serves as a reactive agent and may result in blemish formation. Increasing the level of supplemental copper within a copper source did not increase the number of blemishes observed, though this could be due to the variation observed. The data suggest that CG provides a more reactive copper species than CA and CS at both supplementation levels. As was mentioned previously, CG is comprised of copper ions chelated to the amino acid glutamate in a 1:1 ratio. The copper source CA is similarly a chelated copper source, but copper ions could be chelated to 1 of 17 different amino acids in the same 1:1 ratio. While this experiment did not identify the mechanism behind the formation of blemishes, it can be inferred that one or more amino acids present in CA has a more protective effect on copper. Other researchers have similarly found that not all chelating agents are equal in their protective effects. For example, histidine content of chickpea peptides used to chelate copper affected lipid oxidation (Torres-Fuentes et al., 2014). Understanding the mechanics of why CG was more reactive than CA could aid in identification of a chelated copper source that reduces black blemish formation in canned pet foods. It is possible that copper chelation with one or some of those more protective amino acids could reduce blemish development. This could be explored further in future experimentation.

Canned pet foods are not the only food products to exhibit black spots. Potatoes develop black spots and discoloration when physically damaged, but this has not been linked to copper content (Weaver et al., 1968). Blemishes described as black stippling (Brodrick, 1970) and russeting (development of brown areas; Jones et al., 1994) were documented in citrus and apples, respectively, after the application of copper fungicides to control a disease called black spot. These blemishes were dependent on the amount and type of copper fungicide used, with increased application resulting in more blemishes (Brodrick, 1970). Use of a fungicide containing copper oxychloride resulted in more russeting compared to no fungicide application, while no difference was observed between a copper hydroxide fungicide and no fungicide (Jones et al., 1994). Similar results were observed with the application of fungicides to prevent black spot in Navel oranges. Application of fungicides with either copper ammonium acetate, copper oxychloride, copper hydroxide and ferric chloride, or cuprous oxide all reduced the severity and incidence of black spot. Additionally, the severity of damage to the rind was greater for copper oxychloride fungicide than all other copper-containing fungicides (Miles et al., 2004). These results agree with the present experiment, which found that copper form affected the number of black blemishes observed in canned pet food.

Another mechanism to consider is the development of sulfide black and brown in canned meat. In that instance, the oxidation of sulfur-containing amino acids (methionine and cysteine) due to thermal processing produced hydrosulfide and hydrogen sulfide that interacted with iron and tin from the can wall to form iron sulfide and tin sulfide (Kontominas et al., 2006; Dantas et al., 2014). These compounds created visible black and brown spots, respectively. However, these spots are only found on parts of canned meat that directly touched the can wall and were not associated with copper. Spots in the present experiment were also found on product surfaces that were not in contact with the can wall. It is possible that thermal processing caused copper ions to dissociate from the respective ligand and form copper sulfide, resulting in a dark spot. If this were the case, filling cans at lower temperatures and/or thermal processing at higher temperatures for shorter periods of time may help minimize this spotting (Kontominas et al., 2006). In future experiments, it would be prudent to measure iron and tin concentrations of raw and processed canned pet foods to determine if either metal ion was leaching into the food from the can wall. Higher levels of these minerals were found in tuna, soy oil, octopus, and an aqueous brine that were rejected for can wall imperfections (Kontominas et al., 2006). Traditional methods of methionine and cysteine quantification in animal feed would not be appropriate to directly determine methionine or cysteine oxidation as these methods oxidize all methionine and cysteine (AOAC International, 2012). This makes it impossible to determine if methionine and/or cysteine oxidation was affected by copper supplementation. Instead, oxidized methionine and cysteine could be calculated by subtracting available methionine or cysteine from the total methionine or cysteine, respectively. Methods for available methionine and cysteine utilize enzymatic hydrolysis and reactions with either sodium nitroprusside or 5,5ʹ-dithiobis-2-nutrobenzoic acid (Pieniaąźek et al., 1975). These reactions generate forms of nonoxidized methionine and cysteine, respectively, that can be detected by spectrophotometry with minimal interference from other amino acids.

Additional future work should address pet owner acceptability and perception of thermally processed pet foods with different copper sources at different levels. There is limited published research addressing whether or not consumers can detect color differences in meat products and what they prefer. However, consumers most liked the color of cooked broiler chicken breast meat with higher L* and lower b* values (Ncube et al., 2018). This suggests that pet owners may also prefer lighter and less yellow products, but this has not been confirmed with canned pet foods. Even though smaller shifts were seen here compared to previous literature, supplementation levels should remain at practical values to simulate commercial production of thermally processed canned pet foods. A panel of pet owners should identify blemishes and determine if there is an acceptable blemish size and/or number of blemishes in a product. Another important aspect of this challenge is whether or not storage time has an effect on vitamin E content and visual characteristics of copper-supplemented canned pet food. This could be investigated in a future experiment by storing cans of pet food after production and sampling at appropriate time points.

## CONCLUSIONS

This research observed that vitamin E, redness (a*), yellowness (b*), and black blemish development were affected by copper supplementation. Supplementation level influenced vitamin E content, where CG6 and CS6 contained more vitamin E than CA6, but CS12 contained more vitamin E than CG6 and CA6. Additionally, vitamin E content of CS12 was not different from NC, the treatment without supplemental copper. Color of CG6 was most similar to NC, especially in terms of redness, whereas CA6 and CS6 were less red. Redness and yellowness were affected by the concentration of the copper supplementation; increasing the level of copper supplementation within the same source reduced red and yellow hues except for the yellowness of CA6 vs. CA12. However, CG treatments contained more total and large blemishes than CA or CS within the same supplementation level except for the similar levels of total blemishes between CG12 and CA12. Future research should focus on the mechanism behind this reaction by measuring markers of protein oxidation before and after thermal processing. Such markers could include protein solubility, carbonyls, and available methionine and cysteine. This experiment determined that copper supplementation affected color and blemish development. However, it is not known if a pet owner could identify these differences and determine if they were acceptable or not or if storage time plays a role in the development of visual changes and vitamin E content. This important information is valuable to further address this problem.
